# Association of Picky Eating with Weight and Height—The European Longitudinal Study of Pregnancy and Childhood (ELSPAC–CZ)

**DOI:** 10.3390/nu14030444

**Published:** 2022-01-19

**Authors:** Marketa Grulichova, Daniela Kuruczova, Jan Svancara, Hynek Pikhart, Julie Bienertova-Vasku

**Affiliations:** 1 Department of Pathological Physiology, Faculty of Medicine, Masaryk University, 601 77 Brno, Czech Republic; marketa.grulichova@gmail.com; 2 Research Centre for Toxic Compounds in the Environment (RECETOX), Masaryk University, 601 77 Brno, Czech Republic; 369088@mail.muni.cz (D.K.); svancara@iba.muni.cz (J.S.); h.pikhart@ucl.ac.uk (H.P.); 3 Department of Food Technology, Mendel University, 601 77 Brno, Czech Republic; 4 Research Department of Epidemiology and Public Health, University College London, London WC1E6BT, UK

**Keywords:** picky eating, ELSPAC cohort, cohort study, Czech Republic

## Abstract

Objective: This study aimed to evaluate whether preschool children identified as picky eaters showed differences in anthropometric characteristics (weight and height) from their non-picky peers at 15 years of age. Design: This study was performed among the cohort members of the EL- SPAC–CZ study, a longitudinal study of pregnancy and childhood. The analysis included 2068 children (997 girls and 1071 boys) followed between births and 15 years of age. Picky eaters were identified at 1.5, 3, and 5 years of age. Anthropometric characteristics were measured at 15 years of age (15 years). Results: Picky eaters (*n* = 346; 16.7%) had a lower weight and height than non-picky eaters (*n* = 1722; 83.3%) at 15 years. This difference in weight and height was maintained after controlling for sex of the child, birth weight, birth length, maternal education, family structure at 15 years, and maternal age at childbirth. The picky children were on average 2.3 kg lighter and 0.8 cm shorter than non- picky children at 15 years. Conclusions: Persistent picky eating in preschool children is related to lower weight and height at 15 years of age in ELSPAC–CZ study.

## 1. Introduction

Picky eating (also called a ‘fussy’, ‘faddy’, ‘selective’ or ‘choosy’ food diet) is a common situation in preschool children [1,2] and is classified as part of feeding difficulties [2]. One of the typical features of picky eating is neophobia. Neophobia is characterized by consumption of only favorite foods, unwillingness to try new foods, rejection of certain types of foods, and avoidance of certain textures or amounts of foods [2,3,4]. The rejection of certain food textures may also be affected by the realm of the subjective flavor and the feelings of disgust with food after previous negative experiences [5]. Picky eaters generally require specific procedures of food preparation, they express stronger dislikes and likes for food, and they often have negative emotions (tantrums) when denied certain foods [6]. As for the definition, there is no single definition of picky eating, and there are substantial differences in the methods of assessment used within various studies [2]. The reported prevalence of picky eating was within a wide range in various studies. Tharner et al. reported a prevalence of 5.6% [7], Micali et al. reported 7.3% [8], and Machado et al. found a prevalence of 25.1% [9], whereas Carruth et al. found picky eating ranging from 17% to 47% for males and 23% to 54% for females [10], and Xue, Lee et al. reported a prevalence of 59% [11]. The current consensus is that the development of picky eating is influenced by range of factors including the absence or short duration of exclusive breastfeeding and early introduction of complementary foods (before six months) [12], genetic factors [13], personality factors [5], parental practices and feeding styles [5], paternal controlling feeding [14], or other pressures to eat [5,15,16]. Overall, feeding is considered to be influenced by a highly complex interplay between many factors with varying levels of parental control over the process [13]. It has also been reported that the picky eaters consume fewer food items and have less or inferior variety in their diet than non-picky eaters [2,6,13,17]. Some of the studies also found that picky eaters ate fewer calories than non-picky eaters. Specifically, less total energy, less protein, and fewer total fats were consumed, and picky eaters had a smaller intake of fruit and vegetables, meat, and other protein sources [7,10,18] and sweets [16]. Moreover, the picky eaters were found to have a lower mean intake of carotene, iron, and zinc [19]. There is evidence that picky eaters have a lower weight, height, and BMI than non-picky eaters [1,6,20,21]. Picky eating led to a higher risk of being underweight [22,23,24]; Dubois et al. found picky-eaters to be even twice as likely to be underweight than non-picky eaters [25]. Some studies, on the other hand, found an association with the risk of the development of obesity [22,26,27]. There are very few longitudinal studies focusing on measures of growth in picky children. The long-term effects of picky eating on growth and body composition in the pubescent have received even less attention [28]. Furthermore, there is no evidence about this topic from the Central-European region.

The present study aimed to explore how picky eating in preschool children in Czech Republic, one of the countries of the Central-European region, is longitudinally related to their anthropometric characteristics (weight and height) at the age of 15 years.

## 2. Methods

### 2.1. Study Design

The European Longitudinal Study of Pregnancy and Childhood (ELSPAC) is a population-based study examining the effects of biological, psychosocial, economic, and environmental factors on health and development of children from the prenatal period to adulthood. The original intention of the World Health Organization Regional Office for Europe in 1985 was to collect data from 40,000 children across Europe [29]. Bristol University initially coordinated the project Avon Longitudinal Study of Pregnancy and Childhood (ALSPAC) [30] and protocol development, including follow-up planning and questionnaire design, which was organized by the ALSPAC team. The ELSPAC study (European Longitudinal Study of Pregnancy and Childhood) was established in eight independent centers based in the UK, former Czechoslovakia, Greece, Ukraine, and Russia (the latter two were initially part of the USSR), according to the original ALSPAC protocol (including follow-up planning and questionnaire design).

Data analyzed in this article came from the Czech part of ELSPAC study, which included eligible mothers from the Brno and Znojmo regions of the Czech Republic. All mothers were expected to deliver between 1 April 1991 and 30 June 1992. Out of 5151 children originally enrolled, about 60% of subjects were retained throughout early childhood [31]. Self-reported questionnaires collected the data for participants and included records of demographics, lifestyle, dietary habits, partnership, life attitudes, life events, social factors, and environmental exposure. The Czech ELSPAC study also examined socioeconomic changes related to the societal transformation after the fall of Communism in 1989 [31,32].

### 2.2. Analytical Sample

The analysis included a total of 2068 children who had complete anthropometric data, data on maternal education and family structure, and data about picky eating. In this analytical sample, there were 346 picky eaters and 1722 non-picky eaters. It has to be noted that the classification of children to picky eaters/non-eaters groups is still somewhat subjective.

### 2.3. Dietary Assessment and Anthropometric Measurement

Data were obtained from a questionnaire and anthropometric measurements at three time points for picky eating and one time for anthropometric measurement. Mothers were asked to fill in the questionnaire about eating habits of their children at 1.5 years, 3 years, and 5 years. A single question (the same at all three time points) was used to assess picky eating: “Did it happen anytime in the last year that your child was choosy about their meal? (a) No, it did not happen; (b) Yes, but I did not worry about it; (c) Yes, and I was a little worried about it; (d) Yes, and I was very worried about it”. The children were defined as “picky” if the mothers chose the option (c) or (d) for at least two out of the three investigated time points. The pediatricians performed standardized anthropometric measurements (weight and height) at 15 years of age. Body weight and height were used as dependent variables, while picky eating, height at 15 years (for the analysis of body weight), sex of the child, birth weight, birth length, maternal and paternal height, maternal education, the family structure at 15 years, and maternal age at childbirth were used as independent variables.

### 2.4. Statistical Analysis

Statistical analysis was performed using statistical software R (version 3.6.3) [33]. First, the descriptive characteristics of the sample were calculated, followed by the exploration of relationships between individual variables. To determine the relationship between picky eating and anthropometric characteristics, a two-sample Welch’s *t*-test was used. Afterward, linear models were constructed in order to include potentially confounding variables.

Model 1 describes the relationship between weight/height at 15 years (dependent variable) and picky eating (independent variable).

Model 2 for weight extends the previous model by adding the following covariates: height at 15 years, birth weight, and sex of the child. Model 2 for height extends the first model by adding birth length and sex of the child.

Model 3 for weight/height further extends previous models by including confounders such as maternal education, the family structure at 15 years, and maternal age at childbirth.

Models 2b and 3b add maternal and paternal height as confounders to corresponding models.

Finally, to further explore the effect of variables from Model 3, we additionally assessed potential interactions between sex, maternal education, and family structure at 15 years and picky eating.

Furthermore, two types of sensitivity analyses were performed. The first one was to ensure that whether the child was born from a singleton or twin pregnancy did not affect the results. The second sensitivity analysis focused on our definition of picky eating. To elucidate this, we narrowed down the sample to strict picky eaters (reported as picky by the mother at all three time points) and strict non-picky eaters (reported as non-picky at all three time points), and subsequently, we fitted Models 1, 2, and 3.

The descriptive characteristics were also calculated for subjects excluded from the sample to estimate the effects of attrition. A Pearson’s Chi-squared test was used to determine the difference between excluded and included subjects.

## 3. Results

### 3.1. Descriptive Results

A total of 2068 children were included in the analysis, with 997 girls and 1071 boys. A total of 346 subjects (16.7%) were identified as picky eaters based on our criterion of picky eating at two of three time points, and 1722 (83.3%) of the children were classified as non-picky eaters. The descriptive sample characteristics of the picky and non-picky children are presented in Table 1.

### 3.2. Association of Picky Eating with Weight and Height

We found significant differences in body weight and height between picky and non-picky children (Table 2). The picky children had significantly lower weight and height than non-picky children at the age of 15. Anthropometric characteristic (weight, height, and birth weight) in males and females at 15 years are presented in Table 3.

### 3.3. Regression Models

The initial unadjusted Model 1 showed that the picky eaters had a lower body weight (around 3.3 kg) than non-picky eaters and were shorter (around 1.4 cm).

The extended Model 2 for weight (Table 4) showed that the picky eaters still had a significantly lower weight (around 2.2 kg) than non-picky eaters. A statistically significant association was also found for all three covariates; girls weighed more (around 1.5 kg) than boys, and height at 15 years and birth weight were positively associated with weight. In the Model 2 for height (Table 5), the height of picky eaters stayed significantly lower than the height of non-picky eaters (around 0.9 cm). Girls were significantly shorter than boys (around 8 cm), and the positive association between birth length and height was also significant.

After adding more covariates in Model 3 for weight (Table 4), the results did not substantially change; picky eaters had a significantly lower weight (around 2.3 kg) than non-picky eaters. All covariates, except for maternal age at childbirth, were significantly associated with weight. Higher height and birth weight were associated with higher weight; girls had a higher weight (around 1.6 kg) than boys. Children of mothers with a highest secondary education achieved had a lower body weight (around 1.1 kg) than children of mothers with primary school; similarly, children of mothers with university education had a lower body weight (around 2.3 kg) than those of mothers with primary education. Furthermore, children from single-parent families had a higher weight (around 2.1 kg) than children from original biological families where both parents were present.

Similarly, in Model 3 for height (Table 5), picky eaters were significantly shorter than non-picky eaters (around 0.8 cm). The height was still positively associated with birth length, and girls were shorter than boys (around 8 cm). There was also an association between height and maternal education: children of mothers with an elementary education were significantly shorter than the children of mothers with a high school education (around 1.3 cm) and children of mothers with a university education (around 1.9 cm). No significant association between height and family structure nor maternal age at birth was found.

Adding parental height and weight to the models had very little effect on the association between picky eating and height/weight at 15 years. Picky eaters had, on average, 2.6 kg lower weight and 0.7 cm lower height compared to non-picky eater in these models (shown in the Supplementary Tables S1 and S2). All other associations from the previous models were virtually unchanged. No significant interactions between sex, maternal education, and family structure at 15 years and picky eating were identified, and they were thus excluded from presented results.

Excluding twins from the analysis did not have any effect on the results: both results for height and weight at age 15 remained virtually unchanged. When comparing those classified as strict picky-eaters and strict non-picky eaters, the effect of picky eating in the models for weight was more pronounced. Picky eaters were, on average, 4 kg lighter than non-picky eaters when adjusting for the same variables as in Model 3. With stricter criteria, models for height showed a weaker effect of picky eating.

## 4. Discussion

This longitudinal study investigated the influence of preschool picky eating on body weight and height status at the age of 15 in a cohort of children from the ELSPAC–CZ study. The prevalence of picky eating in the ELSPAC–CZ study was around 16.7%. A wide range of prevalence of picky eating was reported previously by Taylor et al. [2]. Therefore, it is difficult to compare the results of the prevalence of picky eating across various studies. There are huge methodological differences in the presented measurements [34], and uniform methodology to investigate the basis of picky eating is missing. The picky eating status introduced in this study was established based on a single question from the questionnaire completed by the mothers who reported on their children’s feeding behaviour. This approach is similar to the method previously used in the ALSPAC [2] or other studies [6,15,35].

To our knowledge, there have been very few longitudinal studies focusing on growth in picky children. Our analysis included 2068 children (girls and boys), and the picky eaters were identified at 1.5 years, 3 years, and 5 years, and anthropometric data were measured at 15 years of age. We found that the picky eaters had a lower weight and height than non-picky eaters. Specifically, the adjusted models showed negative associations with weight (the picky children were about 2.3 kg lighter than non-picky children) and negative associations with height (the picky children were about 0.8 cm shorter than non-picky children). This result remained significant even after adjustment for various covariates. Such an observation is well in line with the results of the recent analysis of the longitudinal study ALSPAC [28]. The ALSPAC analysis included 7420 children [2] (girls and boys) and identified the picky eaters at three years of age, and weight and height were measured at seven time-points at ages 7–17 years. The ALSPAC results showed evidence of strong negative associations with height in boys and girls (the picky children were about 1.5–2.0 cm and 1.0–1.5 cm shorter than non-picky children) as well as evidence of negative associations with weight in boys and girls (the picky children were about 1.5–2.5 kg and 1.0–1.5 kg lighter than non-picky children) [28]. Similar findings were also observed in the longitudinal study by Berger et al., which included 181 participants (non-Hispanic white girls) [20]. In their study, children were assessed biannually from ages 5 to 15 years and had lower BMIs than non-picky children at every age point from 5 to 15 years of age [20]. Despite similar results, the study by Berger et al. [20] had some differences when compared to our study, e.g., the study included only girls and used a different methodology of data gathering. The data were collected by completing a specific questionnaire, The Child Feeding Questionnaire. Finally, the study by Berger et al. did not include any potential confounders in its model, in contrast to our study or the ALSPAC study [28]. The other studies presented similar results as our study; however, they examined only weight status (or they used the BMI calculated from children’s weight and height), and the design of these studies was cross-sectional [16,36]. Another longitudinal study reported no association between pickiness and child weight or height [37]; however, the subjects were not of the same age as the subjects in our cohort. This cohort included only a small sample of 71 mothers whose children were participating in the study from 2 to 96 months of age, and the design was different, too: mothers reported on children’s diet through a diet record (12 days total), and they participated in an additional interview about mothers’ behaviors [37].

It is important to mention several methodological aspects of our study. The major strength of this study is its relatively large sample size and longitudinal data collection. Furthermore, it is important that our study brings exclusive evidence from a geographical region (Central Europe) where no previous evidence has existed, and it shows similar findings to those previously found in Western Europe in the ALSPAC study [28] and the study by Berger et al. [20] in the USA. Additionally, the differences in height and weight were persistent in models adjusted for the number of confounding variables. On the other hand, the major limitation is the establishment of a picky-eating status solely based on maternal reports, as the parents of picky eaters could be more likely to be concerned with their offspring’s health and wellbeing, and they could overestimate picky eating of their child.

## 5. Conclusions

In conclusion, persistent picky eating in preschool children is related to a lower weight and height at 15 years of age in the ELSPAC–CZ study. These results are in agreement with findings from previous studies; however, this is, to our knowledge, the first study in the Central-European region.

## 6. Key Messages

This study aimed to evaluate whether preschool children identified as picky eaters showed differences in anthropometric characteristics (weight and height) from their non-picky peers at 15 years of age. This study used the data from the European Longitudinal Study of Pregnancy and Childhood (ELSPAC–CZ). Picky eaters were identified at 1.5, 3, and 5 years of age and anthropometric characteristics were measured at 15 years of age (15 years). Differences have been identified between the body weight and height in picky and non-picky children; picky eaters had a lower weight and height than non-picky eaters at 15 years. These findings remained significant after controlling for a range of covariates.

## Figures and Tables

**Table 1:** Sample characteristics.

Variable		Non-Picky (*n* = 1722)		Picky (*n* = 346)		Total (*n* = 2068)		χ^2^ Test of Independence
		N	%	N	%	N	%	*p*-Value
Sex	Female	822	47.74	175	50.58	997	51.79	0.365
	Male	900	52.26	171	49.42	1071	48.21	
Maternal education	Elementary	472	27.41	113	32.66	585	28.29	0.064
	High school	732	42.51	147	42.49	879	42.50	
	University	518	30.08	86	24.85	604	29.21	
Family structure at 15 years	Nuclear family	840	48.78	162	46.82	1002	48.45	0.476
	Stepfamily	114	6.62	31	8.96	145	7.01	
	Single-parent family	158	9.18	32	9.25	190	9.19	
	Unknown	610	35.42	121	34.97	731	35.35	

**Table 2:** Anthropometric characteristic (weight, height, and birth weight) in non-picky and picky eaters at 15 years.

	Non-Picky (*n* = 1722)			Picky (*n* = 346)			*t*-Test
	Mean	SD	Median	Mean	SD	Median	*p*-Value
Height (cm)	170.66	8.31	171.00	169.26	8.49	170.00	0.005
Weight (kg)	60.29	11.56	58.25	56.98	10.16	55.50	<0.001
Birth weight (kg)	3.33	0.47	3.30	3.28	0.50	3.250	0.152

**Table 3:** Anthropometric characteristic (weight, height, and birth weight) in males and females at 15 years.

	Females (*n* = 977)			Males (*n* = 1071)		
	Mean	SD	Median	Mean	SD	Median
Height (cm)	165.87	6.457	166.00	174.67	7.66	175.00
Weight (kg)	56.79	9.65	55	62.48	12.20	61.00
Birth weight (kg)	3.23	0.45	3.25	3.40	0.49	3.40

**Table 4:** Results of linear models for body weight in kilograms at 15 years.

		Model 2			Model 3		
		(*n* = 2068)			(*n* = 2068)		
		Adj. R^2^ = 0.328			Adj. R^2^ = 0.335		
Variable		β	SE	*p*	β	SE	*p*
Picky eater	No	(ref)	-	-	(ref)	-	-
	Yes	−2.18	0.552	<0.001	−2.30	0.550	<0.001
Height (cm)		0.78	0.030	<0.001	0.79	0.030	<0.001
Birth weight (kg)		1.82	0.450	<0.001	1.90	0.450	<0.001
Sex	Male	(ref)	-	-	(ref)	-	-
	Female	1.50	0.484	0.002	1.62	0.484	<0.001
Maternal education	Elementary	-	-	-	(ref)	-	-
	High school	-	-	-	−1.06	0.504	0.036
	University	-	-	-	−2.29	0.568	<0.001
Family structure	Nuclear	-	-	-	(ref)	-	-
	Stepfamily	-	-	-	0.22	0.837	0.789
	Single-parent	-	-	-	2.11	0.738	0.004
	Unknown	-	-	-	0.46	0.456	0.314
Maternal age at childbirth (years)		-	-	-	0.03	0.045	0.487

**Table 5:** Results of linear models for height in centimeters at 15 years.

		Model 2			Model 3		
		(*n* = 2068)			(*n* = 2068)		
		Adj. R^2^ = 0.341			Adj. R^2^ = 0.348		
Variable		β	SE	*p*	β	SE	*p*
Picky eater	No	(ref)	-	-	(ref)	-	-
	Yes	−0.94	0.400	0.018	−0.83	0.399	0.038
Birth length (cm)		0.96	0.069	<0.001	0.92	0.069	<0.001
Sex	Male	(ref)	-	-	(ref)	-	-
	Female	−7.90	0.305	<0.001	−7.94	0.304	<0.001
Maternal education	Elementary	-	-	-	(ref)	-	-
	High school	-	-	-	1.32	0.364	<0.001
	University	-	-	-	1.93	0.410	<0.001
Family structure	Nuclear	-	-	-	(ref)	-	-
	Stepfamily	-	-	-	−0.70	0.607	0.249
	Single-parent	-	-	-	−0.11	0.536	0.832
	Unknown	-	-	-	0.01	0.331	0.976
Maternal age at childbirth (years)		-	-	-	−0.02	0.033	0.623

## Data Availability

Dara is available upon request at www.elspac.cz, (accessed on 18 December 2021).

## Supplementary Materials

The following supporting information can be downloaded at: https://www.mdpi.com/article/10.3390/nu14030444/s1, Table S1: Results of linear models for body weight in kilograms at 15 years with added parental height; Table S2: Results of linear models for height in centimetres at 15 years with added parental height.

## List of Abbreviations

ALSPAC	Avon Longitudinal Study of Pregnancy and Childhood
BMI	Body Mass Index
ELSPAC–CZ	European Longitudinal Study of Pregnancy and Childhood
