# Supplementary material for: Association of Picky Eating with Weight and Height—The European Longitudinal Study of Pregnancy and Childhood (ELSPAC–CZ)
Source: Nutrients. 2022 Jan 19;14(3):444. doi: 10.3390/nu14030444 (PMC8839058; doi:10.3390/nu14030444)
Supplement: Supplementary file 1 [file nutrients-14-00444-s001.zip › nutrients-1539491-supplementary.pdf]

Supplementary table S1 Results of linear models for body weight in kilograms at 15 years with added parental height

		Model 2b			Model 3b		
		**(n=1629*)**			**(n=1629*)**		
		Adj. R^2=0.350			Adj. R^2=0.353		
**Variable**		**β**	**SE**	**P**	**β**	**SE**	**P**
Picky eater	No	(ref)	-	-	(ref)	-	-
	Yes	-2.57	0.620	<0.001	-2.62	0.622	<0.001
Height (cm)		0.87	0.040	<0.001	0.88	0.040	<0.001
Birth weight (kg)		1.39	0.506	0.006	1.50	0.508	0.004
Sex	Male	(ref)	-	-	(ref)	-	-
	Female	2.19	0.573	<0.001	2.26	0.575	<0.001
Maternal height		-0.12	0.044	0.007	-0.10	0.044	0.018
Paternal height		-0.07	0.038	0.068	-0.07	0.038	0.086
Maternal education	Elementary	-	-	-	(ref)	-	-
	High school	-	-	-	-0.67	0.568	0.240
	University	-	-	-	-1.96	0.634	0.002
Family structure	Nuclear	-	-	-	(ref)	-	-
	Stepfamily	-	-	-	-0.61	0.983	0.537
	Single-parent	-	-	-	1.15	0.836	0.171
	Unknown	-	-	-	0.25	0.501	0.619
Maternal age at childbirth (years)		-	-	-	-0.00	0.052	0.957

***439 observations were deleted due to missing data on parental height**

Supplementary table S2 Results of linear models for height in centimetres at 15 years with added parental height

		Model 2b			Model 3b		
		**(n=1629*)**			**(n=1629*)**		
		Adj. R^2=0.543			Adj. R^2=0.545		
**Variable**		**β**	**SE**	**P**	**β**	**SE**	**P**
Picky eater	No	(ref)	-	-	(ref)	-	-
	Yes	-0.64	0.386	0.097	-0.67	0.387	0.083
Birth length (cm)		0.62	0.069	<0.001	0.60	0.069	<0.001
Sex	Male	(ref)	-	-	(ref)	-	-
	Female	-8.27	0.292	<0.001	-8.33	0.292	<0.001
Maternal height		0.44	0.025	<0.001	0.43	0.025	<0.001
Paternal height		0.34	0.022	<0.001	0.34	0.022	<0.001
Maternal education	Elementary	-	-	-	(ref)	-	-
	High school	-	-	-	0.64	0.353	0.069
	University	-	-	-	0.53	0.395	0.181
Family structure	Nuclear	-	-	-	(ref)	-	-
	Stepfamily	-	-	-	0.12	0.612	0.842
	Single-parent	-	-	-	-0.18	0.521	0.735
	Unknown	-	-	-	-0.33	0.312	0.292
Maternal age at childbirth (years)		-	-	-	0.08	0.032	0.018

***439 observations were deleted due to missing data on parental height**
